# The Significance of Halogen Bonding in Ligand–Receptor Interactions: The Lesson Learned from Molecular Dynamic Simulations of the D_4_ Receptor

**DOI:** 10.3390/molecules25010091

**Published:** 2019-12-25

**Authors:** Rafał Kurczab, Katarzyna Kucwaj-Brysz, Paweł Śliwa

**Affiliations:** 1 Department of Medicinal Chemistry, Maj Institute of Pharmacology, Polish Academy of Sciences, Smętna 12, 31-343 Cracow, Poland; katarzyna.kucwaj@uj.edu.pl; 2 Department of Technology and Biotechnology of Drugs, Faculty of Pharmacy, Jagiellonian University Medical College, Medyczna 9, 30-688 Cracow, Poland; 3 Faculty of Chemical Engineering and Technology, Cracow University of Technology, Warszawska 24, 31-155 Cracow, Poland; psliwa@chemia.pk.edu.pl

**Keywords:** halogen bond, XB hot spots, molecular dynamics, dopamine D4 receptor

## Abstract

Recently, a computational approach combining a structure–activity relationship library containing pairs of halogenated ligands and their corresponding unsubstituted ligands (called XSAR) with QM-based molecular docking and binding free energy calculations was developed and used to search for amino acids frequently targeted by halogen bonding, also known as XB hot spots. However, the analysis of ligand–receptor complexes with halogen bonds obtained by molecular docking provides a limited ability to study the role and significance of halogen bonding in biological systems. Thus, a set of molecular dynamics simulations for the dopamine D_4_ receptor, recently crystallized with the antipsychotic drug nemonapride (5WIU), and the five XSAR sets were performed to verify the identified hot spots for halogen bonding, in other words, primary (V5x40), and secondary (S5x43, S5x461 and H6x55). The simulations confirmed the key role of halogen bonding with V5x40 and H6x55 and supported S5x43 and S5x461. The results showed that steric restrictions and the topology of the molecular core have a crucial impact on the stabilization of the ligand–receptor complex by halogen bonding.

## 1. Introduction

Halogen atoms are lipophilic substituents commonly used in the design of novel biologically active molecules to improve their pharmacokinetic profile via, for example, improvement of membrane permeability [1], blood-brain barrier penetration [2] (in the case of Central Nervous System (CNS) active compounds), and metabolic stability [3,4]. During recent decades, it has been widely described that covalently bound halogen atoms (Cl, Br, I) have the ability to form halogen bonds (XBs), acting as donors in a non-covalent directional interaction with a Lewis base as an acceptor [5,6]. This phenomenon is associated with the existence of a σ-hole—a region of depleted electron density, which partially exposes a positive nuclear charge [7,8]. The XB strength is comparable to weak or moderate hydrogen bonds and increases in the order Cl < Br < I. As fluorine atoms are more electronegative and less polarizable than other halogens, they do not form halogen bonds. There is clear evidence indicating that XBs play an essential role in supramolecular systems, liquid crystal engineering, nanomaterials, nanowire formation, and catalysis [9]. According to recent literature, this non-covalent interaction provides new perspectives for drug design, as it appears that the substitution of chemical scaffolds with halogen atoms may significantly improve biological activity [10,11]. Hence, it seems reasonable to deeply analyze the binding pocket of a particular protein target in terms of the presence of potential halogen bond acceptors, thus providing support for the design of novel ligands by indicating in which position(s) halogen atom(s) should be introduced.

Molecular dynamics (MD) simulations of complexes with halogen bonding have been the subject of only a few studies [12,13,14], mainly because it is difficult to properly describe the anisotropy of the electron density of halogen atoms using the available force fields; instead, atoms are defined by the type of atom and their partial charge, and consequently, the possibility of the formation of both halogen bonds (σ-holes) and/or hydrogen bonds (in perpendicular planes to the C–X bonds, where X atoms have electronegative crowns) are not addressed. The first attempt to address the problem of the σ-hole in calculations using force fields was based on the introduction of an off-center positive point charge, an extra site (massless dummy atom) located near the halogen atom [15]. This was successfully applied by Ibrahim et al. in AMBER [16], then Jorgensen et al. in OPLSA-AA [17], as well as by Hobza et al. who introduced the correction called explicit σ-hole (ESH) implemented in the UCSF DOCK molecular docking suite [18]. Another approach, going beyond the partial charge approximation, uses electric multipole expansion (by the sum of multipoles) to describe the anisotropy of the electron density of the halogen atom. However, compared to the off-center positive partial charge approach, the contribution of multipole expansion in molecular dynamics software is still limited [15]. A completely different approach was proposed by Shing Ho et al., whose research group implemented a new potential energy function (ffBXB) to cover the anisotropy of the XB interaction in the AMBER force field [19].

**Figure 1:**
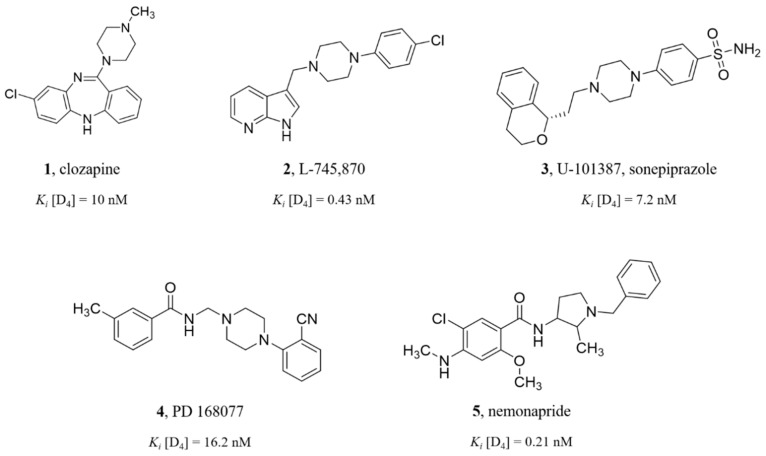
Structures of D_4_ receptor (D_4_R) ligands: a well-known atypical antipsychotic clozapine (**1**) [20], selective antagonists L-745,870 (**2**) and sonepiprazole (**3**) [31] evaluated in clinical trials, the selective agonist PD 168,077 (**4**) [32], and the non-selective agonist nemonapride (**5**) [33].

The dopamine D_4_ receptor (D_4_R) is a subtype of the D_2_-like family belonging to the rhodopsin-like aminergic G protein-coupled receptors (GPCRs). The great attention to D_4_R started in 1991, when it turned out that clozapine (a well-known atypical antipsychotic agent: compound **1**, Figure 1) has a much higher affinity for D_4_ than for the dopamine D_2_ receptor [20]. This discovery suggested refining the dopamine hypothesis of schizophrenia and considering D_4_R involvement in the abovementioned pathophysiological process. It gave hope for the omission of D_2_R receptor activation, which was proposed to be responsible for extrapyramidal side effects and tardive dyskinesias [21,22]. Unfortunately, selective D_4_R antagonists, compounds L-745,870 (2) [23] and sonepiprazole (3) [24], failed in clinical trials in 1997 and 2004, respectively, showing no effects in schizophrenia patients. These results led to a significant decrease in attention focused on D_4_R as a therapeutic target for decades. The situation started to change over the last few years [25] due to the indication of novel, potential non-schizophrenia-like applications of D_4_R antagonists such as in cocaine use disorders [26] and L-DOPA-induced dyskinesias [27]. Moreover, the most recent studies have shown that D_4_R antagonists selectively inhibit the growth of glioblastoma neural stem cells, thus suggesting a place for a new approach to fight with an aggressive and treatment-resistant type of cancer: glioblastoma multiforme (GBM) [28]. Finally, studies performed using an agonist, compound **4** (Figure 1), indicated that D_4_R activation may be a very promising approach to treat potential visual deficits [29]. Nevertheless, many questions remain, and it seems to be necessary to search for novel potent D_4_R ligands. Fortunately, in the last year, the crystal structure of the dopamine D_4_ receptor was determined in complex with the known antipsychotic drug, nemonapride, compound **5** (Figure 1) [30], which allows for a better understanding of the protein–ligand interactions and enables more effective design of new ligands.

Recently, we described a theoretical-experimental approach to define the amino acid hot spots of halogen bonding (i.e., amino acids frequently targeted by halogen bonding in L–R complexes) in another aminergic GPCR class A member, the serotonin 5-HT_7_ receptor [8]. The identification of amino acid hot spots in the binding pocket can significantly support the lead optimization process, directly indicating the position(s) where halogen substituent(s) should be introduced into non-halogenated analogues to improve affinity. To confirm that this paradigm may be applied for the design and/or optimization of ligands of any protein target, XSAR analysis was additionally performed for D_4_R, whose crystal structure has been recently deposited in the Protein Data Bank. The study led to the identification of frequently targeted amino acids by halogen bonding (formed with backbone carbonyl oxygen -c, or with the amino acid side chain -s), in other words, the primary (the GPCRdb generic numbering scheme) V5x40/V193 (c), and the secondary S5x43/S196 (c), S5x461/S200 (c, s), and H6x55/H414 (s). However, the computational workflow included molecular docking to a set of crystal structure conformations of the D_4_ receptor tuned by an induced-fit docking approach. Since the ligand–protein complex is a dynamic system, the docking procedure used is not entirely appropriate to study the significance of particular interactions; for instance, because a given interaction forms and breaks over time. Hence, it was reasonable to perform molecular dynamic (MD) simulations to more rationally define the role of particular interactions in the L–R complex and to verify whether the complex geometry remained stable during the whole simulation.

**Figure 2:**
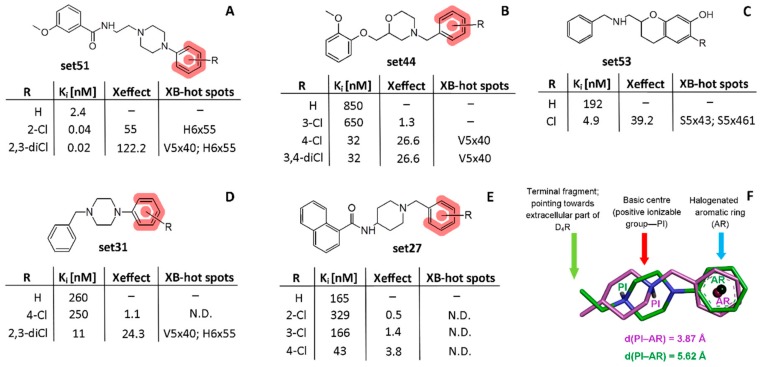
Selected XSAR sets of D_4_R, which were used in the molecular dynamic (MD) simulations study (**A**–**E**). For each set, its chemotype, halogenated derivatives used, their activity towards the D_4_ receptor, Xeffect factor (i.e., the fold of activity change after halogenation), and amino acid hot spots for halogen bonds (XB) that were identified in the previous study [8] on the basis of molecular docking are shown. The alignment of the most frequently observed molecular patterns among XSAR sets (**F**) showing the function of the given fragments with respect to the binding mode and the distance between the positive ionizable group (PI) and the center of the aromatic ring (AR).

As the OPLS3 force field from Schrödinger offers parametrization for halogen bonds, we decided to verify the amino acid hot spots of halogen bonding previously identified for D_4_R by molecular docking of the XSAR library [8]. For this purpose, several D_4_R XSAR sets (Figure 2) representing different chemotypes, which were found to form halogen bonds with hot spots, were used to perform a set of 60 ns-long MD simulations. Trajectory analysis, with particular emphasis on the evolution of the geometry of potential halogen bonds in the studied L–R complexes, were used to confirm the role of the XB hot spots and the importance of this interaction in the stabilization of the L–R complex.

## 2. Results and Discussion

### 2.1. The 5WIU: MD Simulation of the D_4_ Receptor in Complex with Agonist Nemonapride

**Figure 3:**
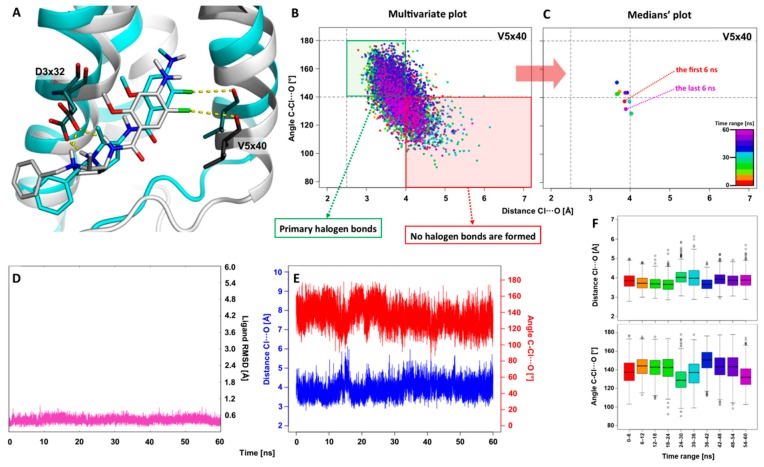
Illustration of the results obtained from the 60 ns-long MD simulation for nemonapride complexed with the D_4_ receptor (PDB ID: 5WIU). Comparison of the starting binding mode (crystal geometry; grey) with the most populated geometry (cyan) obtained from the clustering of the MD trajectory (**A**). The multivariate plot shows the dependence of the distance versus the σ-hole angle for the halogen bond with V5x40 for each trajectory frame, divided into 6 ns-long interval ranges (**B**), and the reduced form of the plot presents the medians of the geometric parameters calculated for given time ranges of the trajectory (**C**). Change in the ligand RMSD during MD simulation (**D**). The change in the distance and σ-hole angle of the halogen bond with V5x40 during the MD simulation (**E**). Boxplots showing the distribution of the distance and the σ-hole angle for each time interval of the MD trajectory (**F**). The labelling of amino acids was based on the GPCRdb generic numbering scheme.

This study started with the analysis of the recently crystallized complex of D_4_R with the agonist nemonapride (PDB code: 5WIU) [30] to verify the formation of halogen bonds in this complex and its stability during MD simulations. The initial geometry was retrieved from the native crystal structure, where the halogen bond with V5x40 was indicated (XB distance d(Cl∙∙∙O) = 3.7 Å, and σ-hole angle = 163.2°). Comparison of the initial binding mode (grey, Figure 3A) with the most populated geometry (cyan, Figure 3A) obtained by clustering of the MD trajectory confirms the maintenance of both key interactions for D_4_R: salt bridge with D3x32 and halogen bond with V5x40. To gain deeper insight into the impact of the halogen bond with V5x40, a multivariate plot showing the dependence of the XB distance Cl∙∙∙O versus the σ-hole angle C–Cl∙∙∙O for each trajectory frame was generated (Figure 3B). The plot area was divided into two regions, in other words, the green region, which represents a geometrically correct halogen bond (the primary XB), and the red region, where no halogen bond formation is observed. The remaining two regions, with adequate parameters of XB distance < 4.0 Å, σ-hole angles < 140°, XB distance > 4.0 Å, and σ-hole angles > 140°, indicate the formation of weak halogen bonds (secondary XB). As previously revealed, this type of XB is not irrelevant due to its frequent occurrence in biological systems [10]. To simplify the multivariate plot (too high data point density) of the XB geometry distribution during the simulation, points corresponding to the medians obtained for the trajectory divided into 6 ns-long intervals were introduced (Figure 3C). In this plot, the fluctuations of XB geometry between primary and secondary XB areas are clearly visible. Additionally, conformational changes of ligands were irrelevant (the RMSD value < 1.0 Å during the whole simulation). Fluctuations of geometric parameters of halogen bond with V5x40 (XB geometry stays in primary XB area for 59% of the simulation time, average distance = 3.8 ± 0.4 Å; σ-hole = 145.0 ± 14.3°; Figure 3E) are significant, which means that XB is formed and broken dynamically, switching from a strong (primary XB area, Figure 3B,C) to a weak interaction (secondary XB area Figure 3B,C). Interestingly, this correlates very well with the low values of the crystal temperature factors (B-factor) for atoms involved in the XB. In summary, the halogen bond with V5x40 is an important factor determining the binding mode of nemonapride with D_4_R. Moreover, the significance of this interaction was recently indicated based on the ab initio FMO calculation in combination with PIEDA analysis [34]. The study showed that XB between V5x40 and nemonapride is mostly dispersion-dependent, which was additional evidence of the hydrophobic nature of XBs.

### 2.2. The Use of XSAR Sets to Explore the Validity of XB Hot Spots

In the next step, the XB hot spots for D_4_R were verified using five XSAR sets, which represent chemically diversified structural patterns (Figure 2A–E). Analysis of the XSAR library (Figure S1) showed that they may be grouped into two classes, differing in the distance between the two key pharmacophore features (Figure 2F, Table S1), in other words, the basic center (PI) and the aromatic ring (AR). The first, and most abundant class for which the PI–AR distance is longer (5.6 Å) contains mainly an arylpiperazine fragment (rarely piperidine), and the second class (less numerous) for which the PI–AR distance is shorter (3.9 Å; benzylpiperidine).

The plots illustrating the change in the distance and σ-hole angle of XB with selected amino acid hot spots during the MD simulation performed for XSAR sets are available in the supplemental information (SI) (Figure S2).

#### 2.2.1. MD Simulations for Sets Showing a Longer PI–AR Distance

**Figure 4:**
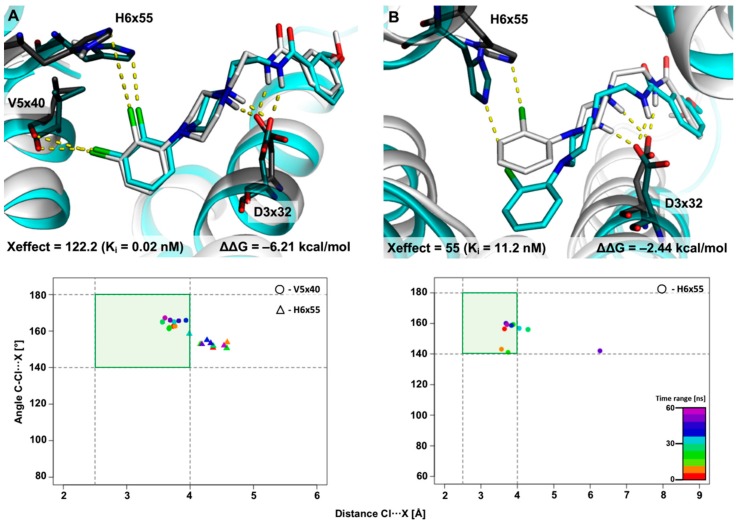
Results of 60 ns-long MD simulation for set51 (**A**: 2,3-diCl, **B**: 2-Cl analogues) with the D_4_ receptor. Comparison of the starting binding mode (induced fit docking; grey) with the most populated geometry (cyan) obtained from clustering of the MD trajectory. The medians’ plot follows the changes in the distances and σ-hole angles for the halogen bond with V5x40 and H6x55.

As a first chemical pattern, two representative XSAR sets containing derivatives with halogenated arylpiperazine fragments were selected. Figure 4 presents the results obtained from the MD simulation for set51, in which the most active is the 2,3-diCl substituted analogue (122-fold increase in activity compared with the unsubstituted analogue, while the 2-Cl derivative shows a 55-fold increase in activity). The initial complexes came from the induced-fit docking of compounds from set51 to the D_4_R crystal structure [8]. The MD trajectory analysis showed stability of the complexes and only slight fluctuations in the geometry during the whole MD simulation (RMSD < 1.0 Å). In complex with the 2,3-diCl analogue (Figure 4A), the halogen bond with V5x40 is strongly involved in the binding mode. The XB geometry stays in the primary XB area for 71% of the simulation time, and the average distance and σ-hole show slight deviations (3.8 ± 0.4 Å; 163.7 ± 8.2°, respectively). Additionally, in this complex, the halogen bond with the H6x55 side-chain shows a noteworthy contribution to the binding mode, however, it should be qualified as a supporting interaction (stays only in the XB secondary area but near the XB primary border). This may be caused by a significantly stronger halogen bond with V5x40, which is crucial for positioning the arylpiperazine moiety in the binding pocket, thus making the XB with H6x55 a secondary interaction (XB geometry stays in primary XB area for 35% of the simulation time, average distance = 4.2 ± 0.5 Å; σ-hole = 153.0 ± 9.5°). To some extent, the proof of this concept is provided by the MD simulation for the 2-Cl analogue (Figure 4B), which shows that the formation of a halogen bond with the H6x55 side-chain is evident; the XB geometry occupies 89% of the simulation time in the XB primary area. The improvement of geometric parameters for the halogen bond and thus an increase in its interaction energy translates into a 55-fold increase in the activity of the 2-Cl analogue in relation to the unsubstituted compound. It should be noted, however, that the XB with the side chain is slightly more ‘blurred’ (average distance = 4.0 ± 0.4 Å; σ-hole = 151.5 ± 20.8°), which is probably caused by the mobility of this side chain.

**Figure 5:**
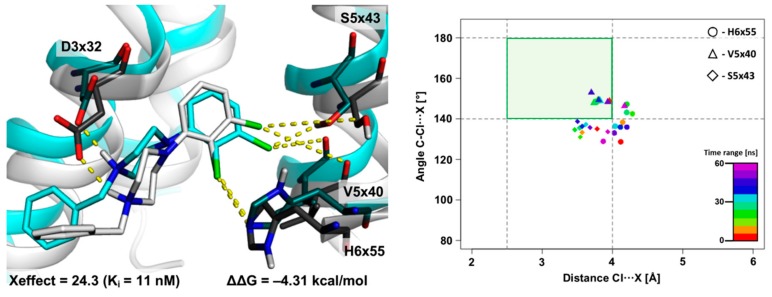
Results of 60 ns-long MD simulation for set31 with the D_4_ receptor. Comparison of the starting binding mode (induced fit docking; grey) with the most populated geometry (cyan) obtained from clustering of the MD trajectory. The medians’ plot follows the changes in the distances and σ-hole angles for the halogen bond with the backbone of V5x40 and the side chains of S5x43 and H6x55.

The next XSAR set31 (containing the halogenated arylpiperazine fragment) showed only slight differences between the geometries of the L–R complexes obtained by the docking and MD procedures (Figure 5). Analysis of the docking-based binding mode indicated that the 2,3-diCl derivative forms a halogen bond with the carbonyl oxygen of V5x40 and side chains of H6x55 and S5x43. MD simulations of this complex confirmed the stability of the binding mode (small dispersion of points in median plot, Figure 5) and the key role of XB with V5x40 (54% of the simulation time the geometry occupies the primary XB area, average distance = 3.9 ± 0.4 Å; σ-hole angle = 151.8 ± 11.6°). With a lesser but still significant impact, an XB with an S5x43 side chain (-OH group) is formed (stays only in the XB secondary area, but near the XB primary border and shows slight deviations from average values; average distance = 3.6 ± 0.5 Å; σ-hole angle = 133.8 ± 14.1°). On the other hand, the geometric parameters of the XB with the H6x55 side-chain show a ‘blurring’ tendency between unfavorable areas of XB formation and the secondary XB area (occupies the primary XB area only 12% of the simulation time with an average distance = 4.1 ± 0.5 Å and σ-hole angle = 130.6 ± 18.6°), which may be caused by the mobility of the H6x55 side chain. It is worth noting that in this case, even as the study describes the use of the same pattern as in XSAR set51, the additional XB with S5x43 is found to contribute to the binding mode. This may be a result of the different influences of diverse terminal fragments (Table S1: the halogenated fragment of arylpiperazines in the same position showed different influences on the Xeffect value), which induces different arylpiperazine positions in the binding pocket through interactions with the extracellular part of the receptor.

#### 2.2.2. MD Simulations for Sets Showing a Shorter PI–AR Distance

The chemical pattern that was analyzed next contained a halogenated benzylpiperidine fragment. There are just a few examples in the XSAR library (Table S1), however, it is interesting to use them in this study as a representation of a structure with a reduced distance between the AR–PI pharmacophore features for D_4_R ligands (compared to arylpiperazine-containing structures).

**Figure 6:**
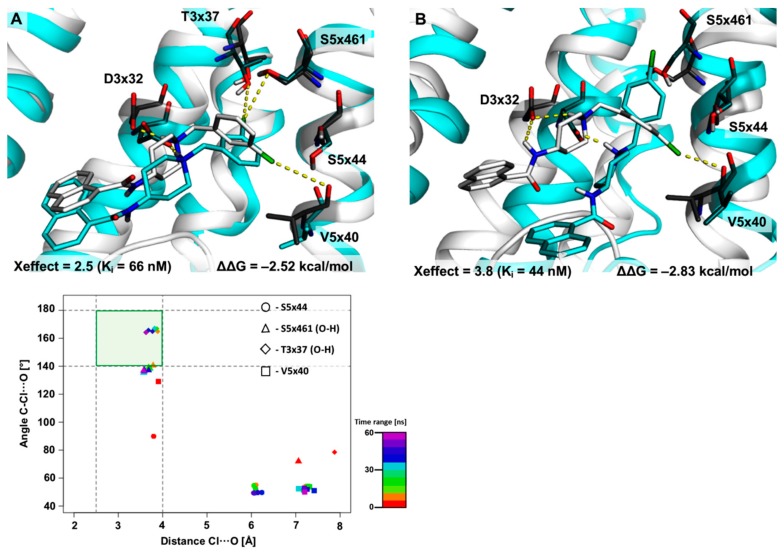
Results of 60 ns-long MD simulation for set27 (**A**: 3-Cl, **B**: 4-Cl analogues) with the D_4_ receptor. Comparison of the starting binding mode (induced fit docking; grey) with the most populated geometry (cyan) obtained from clustering of the MD trajectory. The medians’ plot follows the changes in the distances and σ-hole angles for the halogen bond with backbone of V5x40, S5x44 and side chains of S5x461 and T3x37.

For this purpose, XSAR set27 was selected (Figure 2), for which changes in D_4_R affinity after halogenation are not relevant, and all positions of the benzyl group are substituted with chlorine. Additionally, our previous study [8] showed no interaction via halogen bonds for derivatives within this set, they were labelled as hydrophobic. Interestingly, all three MD simulations for the 2-Cl analogue indicated a significant destabilization of the starting complex, the ligand lost a key interaction (a salt bridge with D3x32 is broken, mainly as a result of inversion at the basic center), which refers to a tendency in activity changes (see Figure 2). MD simulations for the 3-Cl analogue (Figure 6A) show that the secondary halogen bonds identified in the initial complex (i.e., with V5x40 and S5x44) are broken just after the first few nanoseconds of the simulation, and the complex switches to different binding mode, in which new, stable halogen bonds are formed with T3x37 (78% of simulation time in the primary XB area, average distance = 3.7 ± 0.5 Å; σ-hole angle = 165.1 ± 20.4°) and with S5x461 (mainly occupies the secondary XB area, average distance = 3.6 ± 0.8 Å; σ-hole angle = 136.9 ± 16.2°). Finally, in the case of the 4-Cl derivative, the geometry of the initial complex changed rapidly, and the ligand accommodated a new binding mode in which no relevant halogen bond was observed (Figure 6B). The performed MD simulations for this XSAR set indicated a lower preference of the benzylpiperidine scaffold to adopt a conformation in which the halogen atoms are exposed towards V5x40 and/or H6x55, and forcing deeper penetration into the binding pocket may be associated with the possibility of creating new XB contacts (in this case with T3x37).

**Figure 7:**
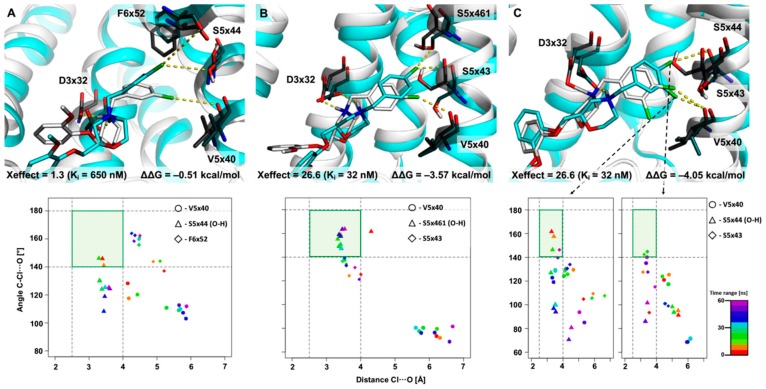
Results of 60 ns-long MD simulation for set44 (**A**: 3-Cl, **B**: 4-Cl, **C**: 3,4-diCl analogues) with the D_4_ receptor. Comparison of the starting binding mode (induced fit docking; grey) with the most populated geometry (cyan) obtained from clustering of the MD trajectory for 3-Cl (**A**), 4-Cl (**B**), and 3,4-diCl analogues (**C**).

The XSAR set44, which contains a halogenated benzylmorpholine fragment, was used as another example with a decreased distance between the pharmacophore features of AR–PI (Figure 7). The previous study [8] indicated that the 3-Cl derivative does not form an optimal halogen bond (geometric parameters for the closest V5x40 are in an unfavorable area for an XB; Figure 7A). During the first 18 ns of MD simulation, the initial geometry changes and the 3-Cl-benzyl moiety penetrates deeper into the binding pocket. As a result, the secondary XBs with the S5x44 side chain (average distance 3.4 ± 0.4 Å; σ-hole angle = 124.3 ± 17.9°) and F6x52 backbone (average distance = 4.4 ± 0.5 Å; σ-hole angle 159.4 ± 13.2°) are formed. For the 4-Cl analogue, the binding mode changed at the beginning of the MD simulation (Figure 7B); the 4-Cl-benzyl fragment penetrated deeper into the binding pocket forming the primary halogen bond with the S5x461 side chain (83% of the simulation time occupied the primary XB area; average distance = 3.4 ± 0.4 Å; σ-hole angle = 156.4 ± 16.8°), and a secondary halogen bond with the S5x43 backbone (average distance = 3.7 ± 0.4 Å; σ-hole angle = 132.6 ± 13.8°). Moreover, the 4-Cl analogue is almost 27-fold more active than the unsubstituted analogue, while the 3-Cl derivative shows only a minor increase in affinity (1.3-fold). The results of the MD simulation indicate that the reason for the substitution-dependent influence on activity is probably concerned with the formation of a halogen bond network with S5x461 and S5x43 by the 4-Cl analogue, thus causing an enhancement in L–R complex stability. The 3,4-diCl derivative shows an identical activity increase as the 4-Cl analogue (Figure 2). Interestingly, substitution with a chlorine atom in position 3 did not influence the activity in comparison to the monosubstituted 3-Cl derivative, for which a slight activity increase was observed. Analysis of the MD trajectories for the 3,4-diCl analogue was therefore partitioned into independent monitoring of the interaction of chlorine atoms in positions 3 and 4 (medians’ plot, Figure 7C). The results did not confirm the formation of stable halogen bonds. Nevertheless, the most populated MD simulation geometry shows halogen bonds with V5x40 and S5x43, which are not located in the primary XB area. Such a picture suggests that L–R complex stability is caused by the rapid formation and breaking of various halogen bonds over time, which might be a result of the greater volume of the 3,4-diCl-benzyl fragment.

#### 2.2.3. MD Simulations for an Uncommon XSAR Set

**Figure 8:**
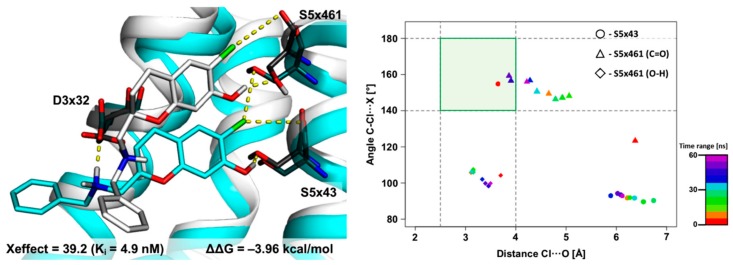
Results of a 60 ns-long MD simulation for set53 with the D_4_ receptor. Comparison of the starting binding mode (induced fit docking; grey) with the most populated geometry (cyan) obtained from clustering of the MD trajectory. The medians’ plot follows the changes of the distances and σ-hole angles for the halogen bond with backbone of S5x43, S5x461 and side chain of S5x461.

Finally, an atypical chemical scaffold (set53), in which the distance between the pharmacophore features PI–AR is identical to those in set51 and set31 but does not belong to the arylpiperazine class, was used. Molecular docking indicates two XB hot spots, namely, S5x43 (with a carbonyl oxygen) and S5x461 (with a side chain). The MD simulations show that the initial complex geometry changed significantly, but key interactions for affinity to D_4_R were maintained (Figure 8). The halogen bond with the S5x43 side chain (placed in the primary XB area) is broken during the first 6 ns and then stays in a non-XB area until the end of the MD simulation. Conversely, the halogen bond with the S5x461 side chain occupies the secondary XB area (average distance = 3.3 ± 0.3 Å; σ-hole angle = 102.9 ± 6.9°), and the formation of a novel interaction with this amino acid is observed (29% of the simulation time stays in the primary XB area; average distance = 4.3 ± 0.6 Å; σ-hole angle 158.5 ± 10.6°).

## 3. Materials and Methods

### 3.1. Structure–Activity Relationship Datasets for Halogenated Analogues 

An algorithm to find all pairs containing halogenated and corresponding unsubstituted structures (called the XSAR library) was developed and used for the D_4_R target in our previous study [8]. To describe the influence of halogenation on the biological activity of the unsubstituted (parent) molecule, the Xeffect parameter was calculated as an activity (extracted from the ChEMBL database during generation of XSAR library) ratio of the parent compound to its halogenated derivative (an Xeffect between 0 and 1 denotes a decrease in the activity upon halogenation and an Xeffect greater than 1 means the fold of activity increased after halogen substitution).

### 3.2. Identification of Halogen Bonding Hot Spots for D_4_R

Privileged amino acids (i.e., hot spots) for halogen bonding for the D_4_ receptor were identified in our previous work using a procedure including the following steps: clustering the halogenated analogues representing each XSAR set, using the centroids of the clusters to tune the D_4_ receptor binding site by an induced-fit docking procedure, QPLD docking [35] of the XSAR library to D_4_R conformations, and determination of the number of halogen bonding interactions with the side chains and carbonyl oxygen atoms of amino acids in the docking poses.

In this study, the GPCRdb generic numbering scheme of amino acids was used [36]. This scheme is based on crystal structures and corrects for helix bulges and constrictions (identified by the structure-based sequence alignments of the recent GPCR crystal structure). For example, V5x40 denotes a valine in transmembrane helix 5, ten positions before the most conserved residue.

### 3.3. Molecular Dynamics Simulations

MD simulations (60 ns) were performed using Schrödinger (Schrödinger, New York, NY, USA) Desmond software [37,38]. The starting L–R complexes selected by molecular docking analysis in our previous study [8] were immersed into a phosphatidylcholine (POPC, 300 K) membrane bilayer and positioned using the PPM web server (http://opm.phar.umich.edu/server.php, accessed 05-01-2019) [39]. Each system was solvated by water molecules described by the TIP4P potential, and the OPLS3 force field parameters were used for all atoms. Additionally, 0.15 M NaCl was added to mimic the ionic strength inside the cell. All simulations were performed on GPU processors, repeating each system three times. Only one trajectory was used to illustrate the results, showing the lowest variation of XB distance and angle values during the simulation. The output MD trajectories were hierarchically clustered into 10 clusters using trajectory analysis tools from the Maestro Schrödinger Suite. For further analysis, the most populated complex was used. Based on obtained trajectories, the mean geometrical parameters of the selected halogen bonds (distance and angle) were calculated using the Simulation Event Analysis tool in Maestro Schrödinger Suite and further analyzed using in-house scripts written in R.

## 4. Conclusions

MD simulations for five XSAR sets were performed to extend our previous study of amino acid hot spots for halogen bonding of the recently crystalized dopamine D_4_ receptor. To select representative sets for simulation, the XSAR library was limited to those sets where halogenated ligands interacted with XB hot spots (i.e., V5x40, S5x43, S5x461, and H6x55). Structural analysis of the reduced XSAR library showed that two types of structural patterns can be distinguished: those containing a longer distance between key pharmacophore features PI–AR (mainly arylpiperazines) and a shorter PI–AR distance (i.e., benzylpiperidines). The two examples of XSAR sets from each group and one set not directly classified in either of the two classes and native agonist nemonapride co-crystallized with D_4_R were used to perform 60 ns-long MD simulations.

Trajectory analysis for XSAR sets containing the arylpiperazine fragment (set51 and set31) confirmed the key role of V5x40 as a hot spot for halogen bonds. In both sets, geometric parameters for XB with V5x40 were in the most energetically favorable areas (simultaneously the conditions of XB distance < 4.0 Å and angle > 140° were fulfilled) for almost the whole simulation time and showed high stability (small standard deviations for the XB length and angle). It is worth noting that the halogenated derivatives in these sets (2,3-diCl in both sets and 2-Cl in set51) formed halogen bonds with H6x55 (i.e., a chlorine in position 2 of the phenyl ring), which was classified as a supporting interaction (also having good geometrical parameters and a small standard deviation during simulation). Moreover, considering that the starting geometry of the ligands in both sets did not change significantly during the simulation, the changes in activity (Xeffect) of the halogenated derivatives in relation to the unsubstituted analogues showed a significant increase, and the above results of the MD simulation can confirm the hypothesis of the privileged role of halogen bonding with V5x40 and supporting (the secondary XB) interaction with H6x55. Additionally, the trajectory analysis for nemonapride confirmed the significant role of the XB bond with V5x40 (the XB geometry and its stability were slightly more blurred). Based on these findings, it can be concluded that the carbonyl oxygen of V5x40 and/or nitrogen of H6x55 can be used as XB anchoring points for designing new D_4_ ligands containing arylpiperazine, piperidine, or an analogous fragment with a comparable PI–AR distance (e.g., set53).

Interestingly, the results of the MD simulations performed for sets representing a second class containing structures with shorter distance between PI–AR pharmacophore features (set44 and set 27) did not confirm the role of halogen bonding with V5x40 (set44), and the starting geometries in the first few ns of the simulation changed; they penetrated deeper into the binding pocket of the receptor. As a consequence, new halogen bonds were formed, mainly with the secondary hot spots S5x43 and S5x461 indicated in the previous study and with T3x37 and F6x52, which had not been observed previously. It should be noted that in this case, the role of the halogen bond is less significant because the Xeffect values for these sets are lower than in the first class (where the XB with V5x40 was evident).

It should be mentioned that the privileged amino acids for halogen bonding in the D_4_ receptor validated in this study have already been identified as important for interactions with endogenous and synthetic ligands. For instance, Cummings et al. [40] performed a number of D_4_ receptor mutations and found the key role of 5x43 and 5x461 serines in the formation of L–R active complexes (mutation to alanine caused a drastic decrease in activity). In identical studies, but for the D_2_ receptor [41,42], the key role of the mentioned serines from helix 5 was also proven, and the multidimensional function of H6x55 in ligand binding was additionally emphasized. The role of the XB with the carbonyl oxygen of S5x43 in the D_2_ receptor was also indicated in MD simulations, which might trigger conformational changes in helix 5 that could consequently modify the overall stability of the ligand–receptor complex [13]. It should be stressed that S5x43, S5x461 and H6x55 are all conserved motifs in the structure of the D_2–4_ receptors.

The results of the MD simulations supported by the experimental data showed that steric restrictions of the receptor and the topology of the ligand’s molecular core have a key impact on the stabilization of the L–R complexes by halogen bonding. The level of enhancement in the activity of the halogenated derivative compared to its unsubstituted analogue depends on the stability of the halogen bond, the type of amino acid hot spot targeted, and the molecular pattern of the designed molecule. Finally, it seems that induced-fit docking may not be sufficient in binding mode analysis of XSAR sets, and thus MD simulations should be used next to study potential XB formation in L–R complexes.

## Supplementary Materials

The following are available online, Figure S1: The XSAR matrix for D_4_R target, Figure S2: The dependency of the change in length and angle of the halogen bonds formed between the analogues from the XSAR library and the selected amino acid of the D_4_ receptor binding site, Table S1: Analysis of the XSAR library molecular scaffolds (chemotypes).
