# Supplementary material for: The Significance of Halogen Bonding in Ligand–Receptor Interactions: The Lesson Learned from Molecular Dynamic Simulations of the D4 Receptor
Source: Molecules. 2019 Dec 25;25(1):91. doi: 10.3390/molecules25010091 (PMC6983170; doi:10.3390/molecules25010091)
Supplement: Supplementary file 1 [file molecules-25-00091-s001.pdf]

Supporting information

The significance of halogen bonding in ligand-receptor interactions – the lesson learned from Molecular Dynamic simulations of the D_4 receptor

Rafał Kurczab[1,*], Katarzyna Kucwaj-Brysz[1,2] and Paweł Śliwa[3]

[1] Department of Medicinal Chemistry, Maj Institute of Pharmacology, Polish Academy of Sciences, Smetna 12, 31-343 Cracow, Poland; kurczab@if-pan.krakow.pl
[2] Department of Technology and Biotechnology of Drugs, Faculty of Pharmacy, Jagiellonian University Medical College, Medyczna 9, 30-688 Cracow, Poland
[3] Faculty of Chemical Engineering and Technology, Cracow University of Technology, Warszawska 24, 31-155 Cracow, Poland

Figure S1. The XSAR matrix for D4R target.

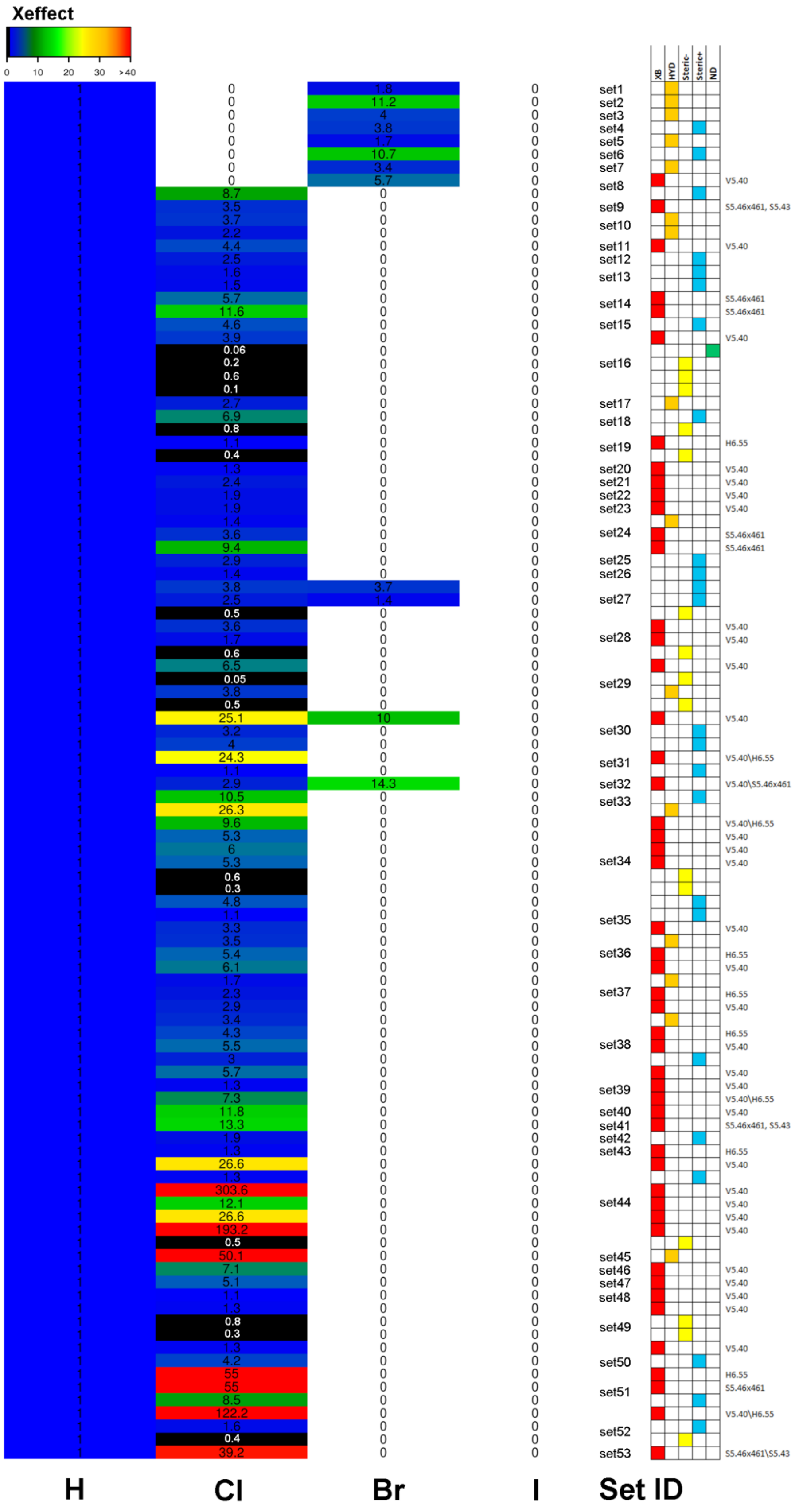

Figure S2. The dependency of the change in length (blue line) and angle (red line) of the halogen bond formed between the analog of the XSAR library and the selected amino acid of the D_4 receptor binding site.

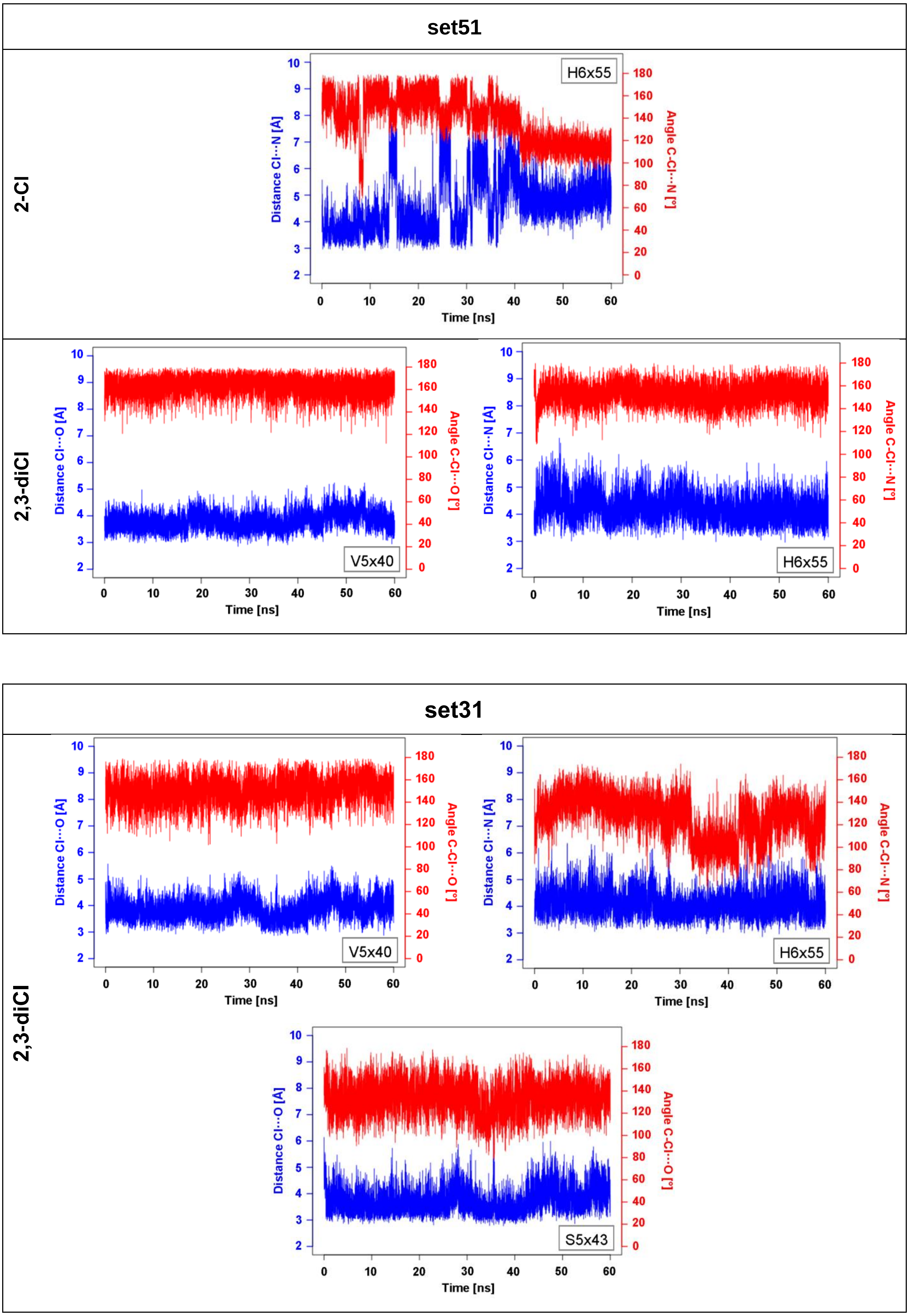

set27

3-Cl

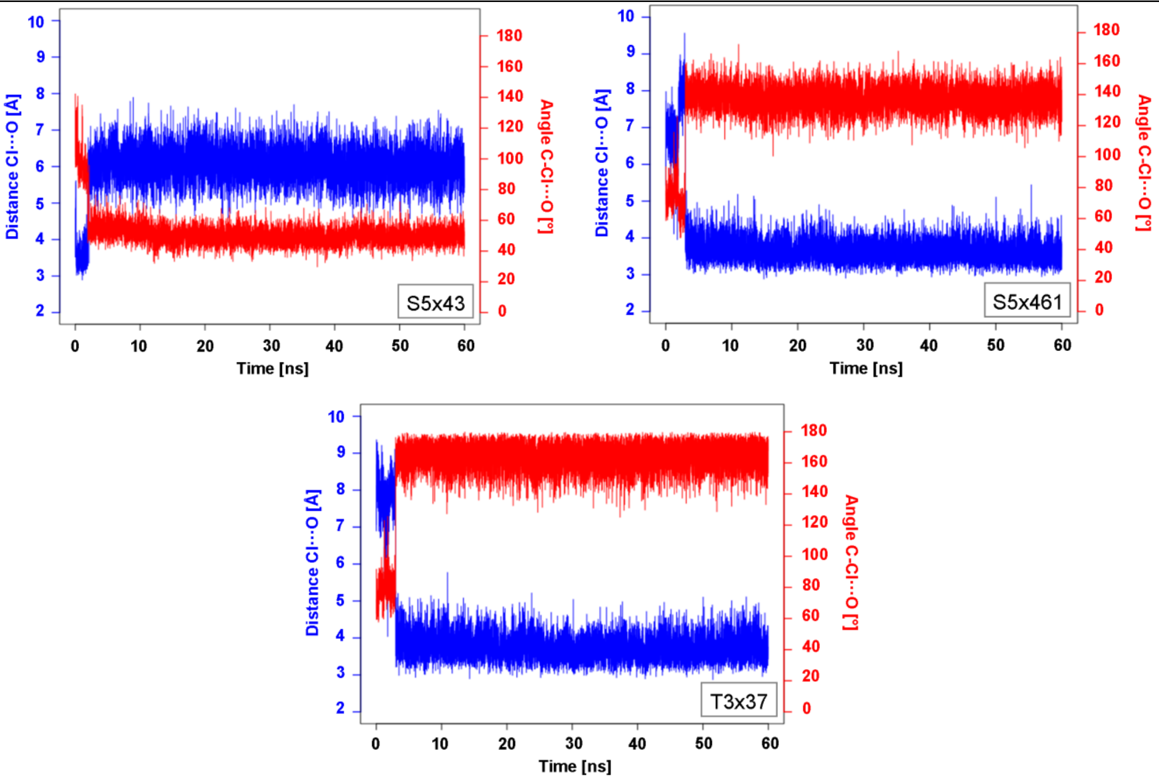

set44

3-Cl

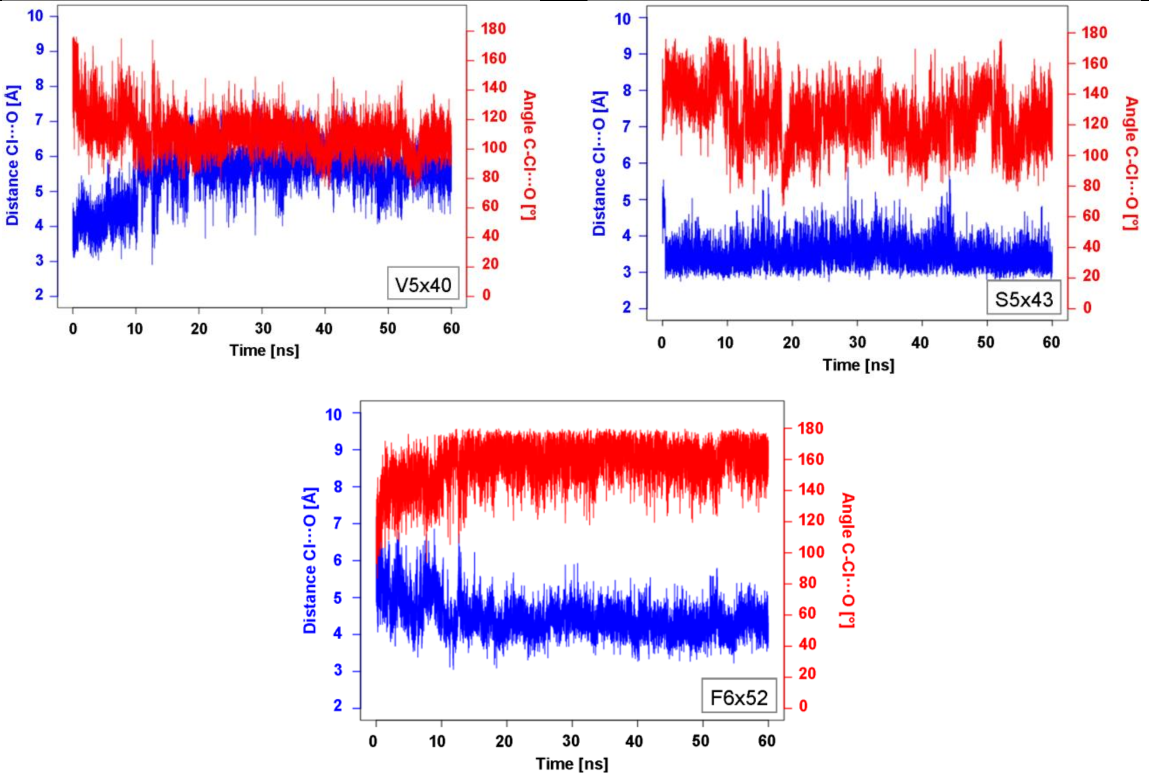

4-Cl

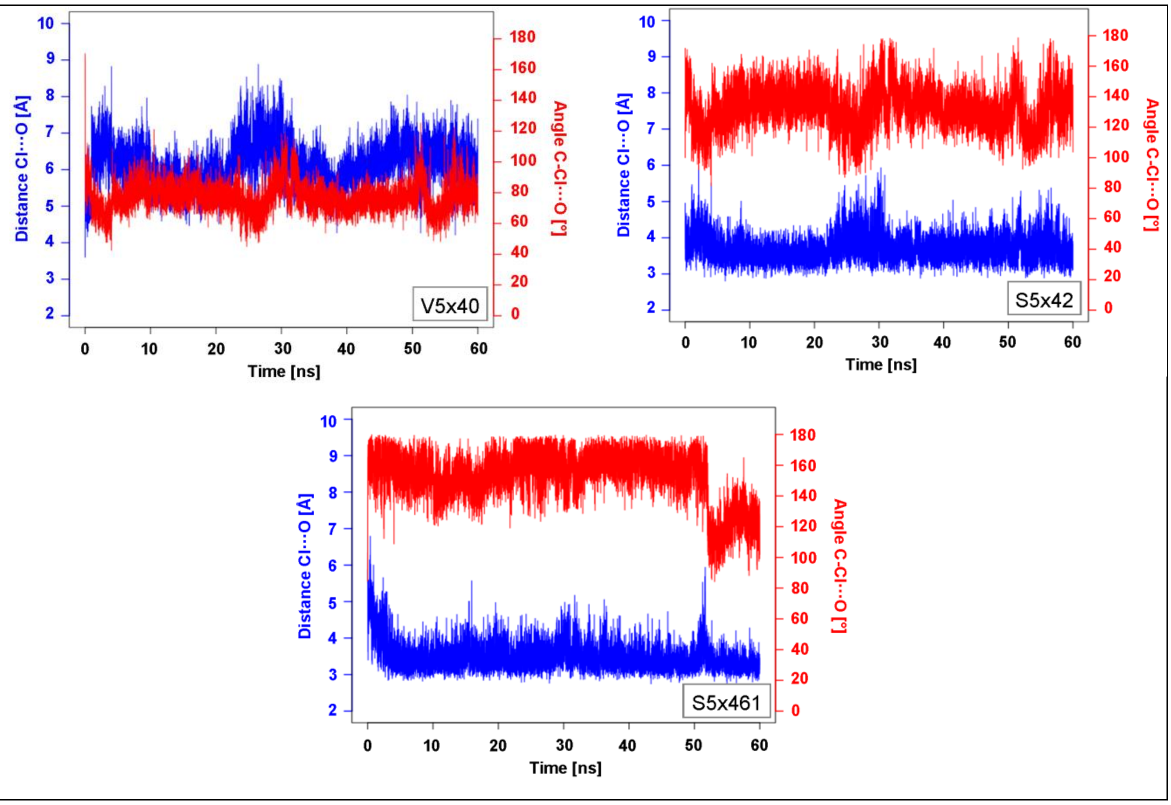
V5x40
S5x42
S5x461
Distance Cl···O [Å]
Angle C-Cl···O [°]
Time [ns]

set53

Cl

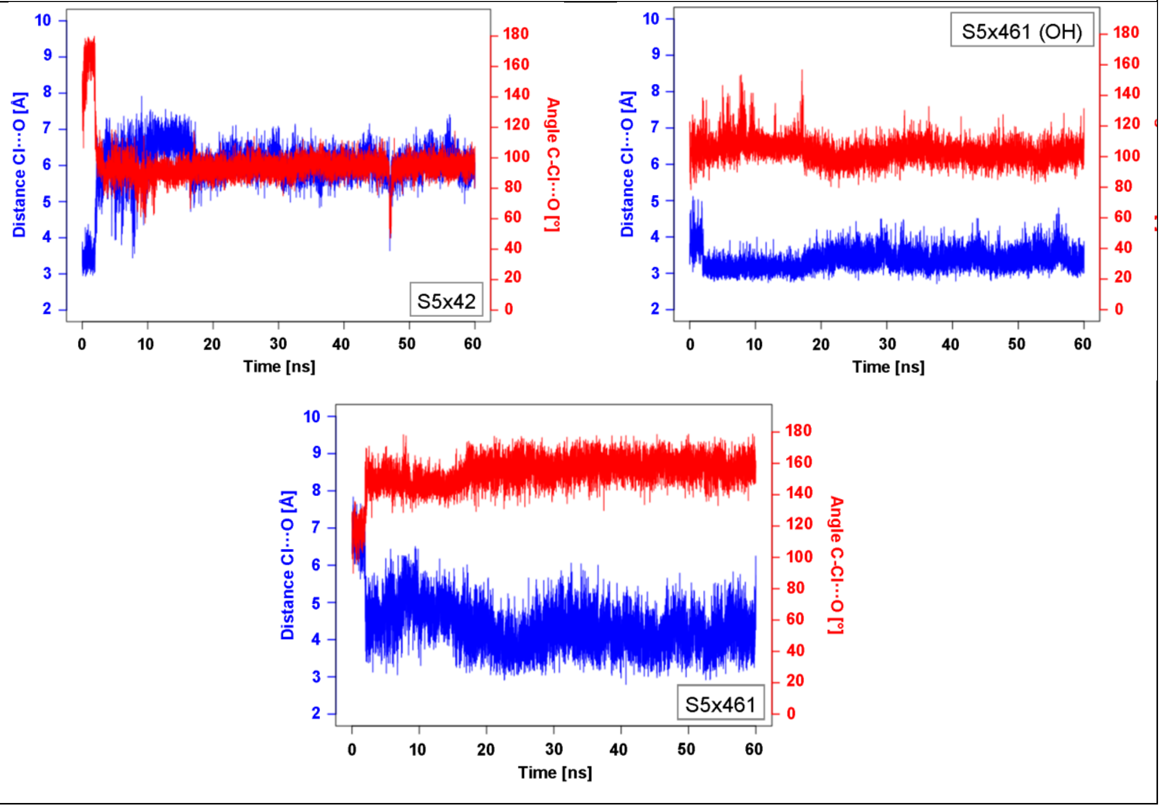
S5x42
S5x461 (OH)
S5x461
Distance Cl···O [Å]
Angle C-Cl···O [°]
Time [ns]

Table S1. Analysis of the XSAR scaffolds.

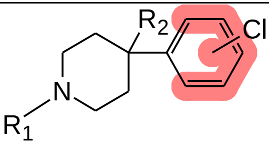

Set ID	R₁	R₂	Xeffect vs. Halogen position		
			ortho	*meta*	*para*
32	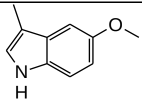	OH	-	-	2.9
52	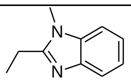	H	-	0.4	1.6

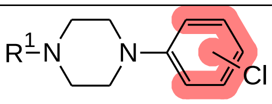

<table>
<tr><th rowspan="2">Set ID</th><th rowspan="2">R₁</th><th colspan="3">Xeffect vs. Halogen position</th></tr>
<tr><th>ortho</th><th>meta</th><th>para</th></tr>
<tr><td>11</td><td>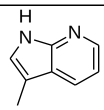</td><td>–</td><td>–</td><td>4.4</td></tr>
<tr><td rowspan="2">19</td><td rowspan="2">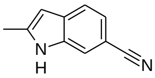</td><td rowspan="2">1.1</td><td>–</td><td>–</td></tr>
<tr><td colspan="2">0.4</td></tr>
<tr><td>20</td><td>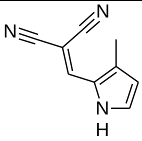</td><td>–</td><td>–</td><td>1.3</td></tr>
<tr><td>21</td><td>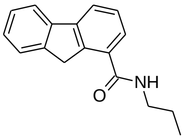</td><td colspan="2">2.4</td><td>–</td></tr>
<tr><td>22</td><td>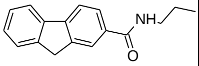</td><td colspan="2">1.9</td><td>–</td></tr>
<tr><td>23</td><td>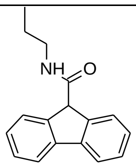</td><td colspan="2">1.9</td><td>–</td></tr>
<tr><td>26</td><td>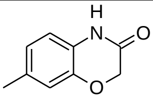</td><td>–</td><td>–</td><td>1.4</td></tr>
<tr><td>30</td><td>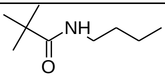</td><td>4.0</td><td>3.2</td><td>25.1</td></tr>
<tr><td>31</td><td>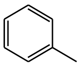</td><td>24.3</td><td>1.1</td><td>31</td></tr>
<tr><td>34</td><td>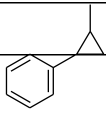</td><td>6</td><td>0.6</td><td>9.6</td></tr>
</table>

<table>
<tr><td rowspan="2"></td><td rowspan="2"></td><td colspan="2">5.3</td><td></td></tr>
<tr><td>–</td><td colspan="2">4.8</td></tr>
<tr><td rowspan="2">35</td><td rowspan="2">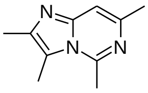</td><td rowspan="2">–</td><td>–</td><td>1.1</td></tr>
<tr><td colspan="2">3.3</td></tr>
<tr><td>36</td><td>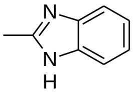</td><td>6.1</td><td>5.4</td><td>3.5</td></tr>
<tr><td rowspan="2">37</td><td rowspan="2">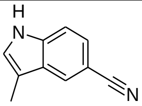</td><td rowspan="2">2.9</td><td>–</td><td>1.7</td></tr>
<tr><td colspan="2">2.3</td></tr>
<tr><td rowspan="2">38</td><td rowspan="2">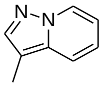</td><td rowspan="2">5.7</td><td>4.3</td><td>5.5</td></tr>
<tr><td colspan="2">3.0</td></tr>
<tr><td>39</td><td>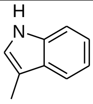</td><td colspan="2">1.3</td><td>7.3</td></tr>
<tr><td>46</td><td>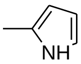</td><td>–</td><td>–</td><td>7.1</td></tr>
<tr><td>48</td><td>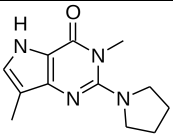</td><td>–</td><td colspan="2">3.4</td></tr>
<tr><td rowspan="3">49</td><td rowspan="3">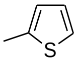</td><td>1.3</td><td>–</td><td rowspan="2">0.3</td></tr>
<tr><td colspan="2">1.3</td></tr>
<tr><td>–</td><td colspan="2">0.8</td></tr>
<tr><td rowspan="2">51</td><td rowspan="2">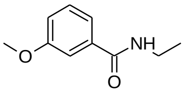</td><td>55</td><td>8.5</td><td rowspan="2">55</td></tr>
<tr><td colspan="2">122</td></tr>
</table>

<table>
<tr><td colspan="7">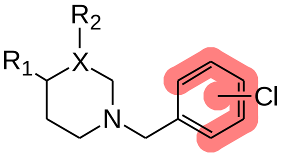</td></tr>
<tr><td rowspan="2">Set ID</td><td rowspan="2">R1</td><td rowspan="2">R2</td><td rowspan="2">X</td><td colspan="3">Xeffect vs. Halogen position</td></tr>
<tr><td>ortho</td><td>meta</td><td>para</td></tr>
<tr><td>27</td><td>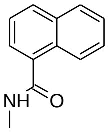</td><td>–</td><td>C</td><td>0.5</td><td>1.4</td><td>3.8</td></tr>
<tr><td>43</td><td>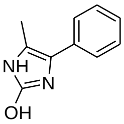</td><td>–</td><td>C</td><td>0.3</td><td>1.3</td><td>0.5</td></tr>
<tr><td>44</td><td>–</td><td>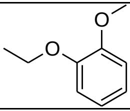</td><td>O</td><td>–</td><td>1.3</td><td>26.6</td></tr>
</table>

					26.6	
47	–	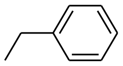	O	–	–	5.1

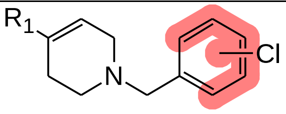

Set ID	R_1	Xeffect vs. Halogen position		
		ortho	*meta*	*para*
14	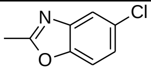	–	–	2.1
16	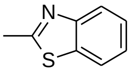	0.1	0.6	3.9
24	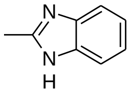	–	–	1.4
